# Enhanced Renal Afferent Arteriolar Reactive Oxygen Species and Contractility to Endothelin-1 Are Associated with Canonical Wnt Signaling in Diabetic Mice

**DOI:** 10.1159/000490334

**Published:** 2018-05-30

**Authors:** Suping Zhang, Qian Huang, Qiaoling Wang, Qin Wang, Xiaoyun Cao, Liang Zhao, Nan Xu, Zhengbing Zhuge, Jianhua Mao, Xiaodong Fu, Ruisheng Liu, Christopher S. Wilcox, Andreas Patzak, Lingli Li, En Yin Lai

**Affiliations:** a Department of Physiology, and the Children’s Hospital, Zhejiang University School of Medicine, Hangzhou,; b Department of Physiology, Quanzhou Medical College, Quanzhou,; c Department of Physiology, School of Basic Medical Sciences, Guangzhou Medical University, Guangzhou, China,; d Department of Molecular Pharmacology & Physiology, University of South Florida College of Medicine, Tampa, Florida,; e Division of Nephrology and Hypertension, and Hypertension Center, Georgetown University, Washington, DC, USA,; f Institute of Vegetative Physiology, Charité-Universitätsmedizin Berlin, Berlin, Germany

**Keywords:** Endothelin-1, Reactive oxygen species, Canonical Wnt signaling, Afferent arteriole, Diabetes mellitus

## Abstract

**Background/Aims:**

Canonical Wnt signaling is involved in oxidative stress, vasculopathy and diabetes mellitus but its role in diabetic renal microvascular dysfunction is unclear. We tested the hypothesis that enhanced canonical Wnt signaling in renal afferent arterioles from diabetic mice increases reactive oxygen species (ROS) and contractions to endothelin-1 (ET-1).

**Methods:**

Streptozotocin-induced diabetes or control C57BI/6 mice received vehicle or sulindac (40 mg·kg^−1^·day^−1^) to block Wnt signaling for 4 weeks. ET-1 contractions were measured by changes of afferent arteriolar diameter. Arteriolar H_2_O_2_, O_2_^.−^, protein expression and enzymatic activity were assessed using sensitive fluorescence probes, immunoblotting and colorimetric assay separately.

**Results:**

Compared to control, diabetic mouse afferent arteriole had increased O_2_^−^(+ 84%) and H_2_O_2_ (+ 91%) and enhanced responses to ET-1 at 10^−8^ mol·l^−1^ (−72±4% of versus −43±4%, *P*<0.05) accompanied by reduced protein expressions and activities for catalase and superoxide dismutase 2 (SOD2). Arteriolar O_2_^.−^ was increased further by ET-1 and contractions to ET-1 reduced by PEG-SOD in both groups whereas H_2_O_2_ unchanged by ET-1 and contractions were reduced by PEG-catalase selectively in diabetic mice. The Wnt signaling protein β-catenin was upregulated (3.3-fold decrease in p-β-catenin/β-catenin) while the glycogen synthase kinase-3β (GSK-3β) was downregulated (2.6-fold increase in p-GSK-3β/GSK-3β) in preglomerular vessels of diabetic mice. Sulindac normalized the Wnt signaling proteins, arteriolar O_2_^.−^, H_2_O_2_ and ET-1 contractions while doubling microvascular catalase and SOD2 expression in diabetic mice.

**Conclusion:**

Increased ROS, notably H_2_O_2_ contributes to enhanced afferent arteriolar responses to ET-1 in diabetes, which is closely associated with Wnt signaling. Antioxidant pharmacological strategies targeting Wnt signaling may improve vascular function in diabetic nephropathy.

## Introduction

Diabetes mellitus (DM) is a microvascular disorder characterized with a progressive decrease in renal blood flow (RBF) although the RBF and glomerular filtration rate (GFR) may increase early in its hyperfiltration phase [1]. The tone of renal afferent arteriole is the key element in regulation of RBF. The arteriolar tone is determined by many factors, two of which are the major contributors to increase the arteriolar tone: apparent oxidative stress in animal renal afferent arterioles[1], and elevated plasma and tissue endothelin 1 (ET-1) levels in animal models and patients with DM [2–4].

Oxidative stress is a shift of balance oxidants versus antioxidants (superoxide dismutase (SOD) and catalase) in favor of oxidants, thereby resulting in excessive reactive oxygen species (ROS), mainly superoxide anion (O_2_^.−^) and hydrogen peroxide (H_2_O_2_), in the tissues. Both O_2_^.−^ and H_2_O_2_ have been implicated in modulating myogenic contraction. O_2_^.−^ promotes normal myogenic contractions [5, 6] whereas H_2_O_2_ has opposite effects [6, 7]. Diabetic animals have increased ROS in renal afferent arterioles [1] and increased responses to ET-1 in microvessels from pancreatic islet [8] and the optic fundus capillary [9]. Studies in penile arteries from insulin-resistant obese rats has demonstrated that O_2_^.−^ enhances contractions to ET-1[10], but the contribution of H_2_O_2_ is unclear.

The canonical Wnt pathway entails signaling through inactivation of glycogen synthase kinase-3β (GSK-3β) that stabilizes the cytosolic β-catenin by inhibition of its degradation. This pathway underlines renal damage from high glucose [11, 12]. Canonical WNT signaling can regulate redox signaling in vasculatures, [13] mitochondrial system [14] and diabetic animal [15]. However, its specific role in regulation of renal microvascular reactivity in DM has not been determined. Therefore, the aim of this study was to investigate whether enhanced canonical Wnt signaling contributes to the increased ROS and thereby to enhanced contractions to ET-1 in renal afferent arterioles of diabetic mice. O_2_^.−^ and H_2_O_2_ in the arterioles were assessed by fluorescence and biochemical determination and from changes in vascular function after incubation with PEG-SOD or PEG-catalase. The specific role of canonical Wnt signaling in regulation of ROS was assessed from the responses to 4 weeks of oral administration of its inhibitor sulindac in streptozotocin (STZ)-induced diabetic mice [16].

## Materials and Methods

### Animals

Male adult C57B1/6 mice (SLAC laboratory animal company, Shanghai, China) with an average weight of 25 g were located under standard conditions with free access to standard pellet chow and tap water. All the experiments were performed with approval from the Institute Animal Care and Ethical Committee of Zhejiang University School of Medicine.

### Induction of STZ-induced diabetic mice model

Diabetes was induced by intraperitoneally injections of Streptozotocin (STZ, diluted in citrate buffer, 0.1 M, pH 4.5) at 70 mg·kg^−1^·day^−1^ for 4 consecutive days. Seven days after STZ injection, mice with glucose levels over 16.7 mmol·l^−1^ were selected for the following experiments. Citrate buffer instead of STZ was set as vehicle control. The food and water intake, body weight and kidney weight were measured in all experimental groups. Plasma glucose level was monitored by a contour glucose meter (Roche, Mannheim, Germany) once a week for 4 weeks. To investigate the effect of Wnt signaling on ROS generation and arteriolar activity, sulindac (40 mg·kg^−1^·day^−1^) or vehicle was administered intragastrically to vehicle control and diabetic mice daily for 4 weeks.

### Isolation and perfusion of renal afferent arterioles

Afferent arterioles were isolated and perfused as previously described [17, 18]. Briefly, an afferent arteriole attached to its intact glomerulus was separated from the renal cortex at 4°C in Dulbecco’s modified Eagle’s medium (DMEM) and transferred to a temperature-regulated chamber on the stage of an inverted microscope (IX71, Olympus, Japan). The afferent arteriole was perfused at 37°C with an array of glass pipettes. The pressure in the tip of perfusion pipette was 60 mmHg. Only one afferent arteriole per animal was used for one experiment. Arterioles with a fast and complete contraction in response to KCl (100 mmol·l^−1^) solution were used in the experiments.

### Measurement of afferent arteriolar diameter

The experimental data were recorded real-time and luminal diameters were measured to determine the effect of vasoactive substances. In all series, the last 30 seconds of the 2 minutes control or treatment period was used for statistical analysis of steady state responses [19]. The protocols were performed (one arteriole for one protocol) as follows: (1) cumulative bath addition of ET-1 (10^−12^ – 10^−8^ mol·l^−1^); (2) preincubation with PEG-catalase (1000 units·ml^−1^,30 min) [6] followed by cumulative bath addition of ET-1; preincubation with PEG-SOD (200 units·ml^−1^,30 min) [19] followed by cumulative bath addition of ET-1; incubation of H_2_O_2_ (10 μmol·l^−1^,15 mins). Contractions of afferent arterioles are presented as relative values of luminal diameters (percentage of the initial diameter).

### Isolation of renal pregiomeruiar arterioles

Isolation of pregiomeruiar arterioles was performed as previously described [19–21]. Briefly, the renal vessels in the anesthetized mice were perfused with 1% iron oxide in physiological phosphate buffered saline (PBS). The perfused segments of renal pregiomeruiar microvessels (interlobular arteries and afferent arterioles) were isolated by using the high-performance magnet. The isolated microvessels were used for the following biochemical analysis.

### Measurement of superoxide and hydrogen peroxide

As described previously,[19] in the perfused individual afferent arteriole, changes in ethidium:dihydroethidium (E:DHE) fluorescence ratio and 6-carboxy-2′, 7′-dichlorodihydrofluorescein diacetate (C-H_2_DCFDA) were used for detection of O_2_^.−^ and H_2_O_2_, respectively. In the isolated pregiomeruiar arterioles, O_2_^.−^ and H_2_O_2_ were analyzed using standard assay kits according to manufactures instructions. O_2_^.−^ or H_2_O_2_ levels were presented as units or μmol per milligram protein.

### Enzymatic activity assay

Endogenous antioxidant enzymes, SOD and catalase, were measured using colorimetric assay kits according to the manufacturers’ instructions, as described previously [19]. Briefly, the isolated pregiomeruiar arterioles were homogenized at 4°C. Enzymatic activities in the supernatants were determined based on its ability to form H_2_O_2_ and degradation rate of H_2_O_2_ for SOD and catalase, respectively.

### Western Blot analysis

Each protein sample was extracted from the isolated pregiomeruiar arterioles of one mouse. The samples were loaded and separated by SDS-polyacrylamide gel electrophoresis and transferred onto polyvinylidene difluoride membranes. The specific proteins on the membranes were probed with specific primary antibodies [22,23] for GSK-3β, phospho-GSK-3β (Ser9), β-catenin, phosphor-β-catenin (Ser33/37/The41), SOD1, SOD2, catalase or β-actin and second horseradish peroxidase-labeled IgG anti-rabbit (or mouse) antibody. The probed bands were visualized by enhanced chemiluminescent substrates (ECL, Thermo Scientific) and analyzed by using Image J software (National Institutes of Health, Bethesda, MD).

### Chemicals and reagents

Reagent sources were as follows: DMEM, sulindac, ET-1, PEG-catalase, PEG-SOD, apocynin (Sigma-Aldrich, St Louis, MO); DHE, C-H_2_DCFDA (Invitrogen Life Technologies, Eugene, Dregon, USA); BCA protein assay kit, H_2_O_2_ assay kit, catalase assay kit and total SOD assay kits (Beyotime, Shanghai, China). O_2_^.−^ assay kit (Jiancheng Bioengineering Institute, Najing, China). Antibody sources were as follows: rabbit anti-GSK-3β, rabbit anti-phospho-GSK-3β (Ser9), rabbit anti-β-catenin, rabbit anti-phospho-β-catenin (Ser33/37/Thr41), rabbit anti-SODl (#2770), rabbit anti-SOD2 (#13194), rabbit anti-catalase (#14097), horseradish peroxidase-labeled IgG anti-rabbit (or mouse) antibodies (Cell Signaling Technology, Beverly, CA, USA) and mouse anti-β-actin (Abeam, Cambridge, MA, USA).

### Statistical analysis

Data were presented as means ± SEM values. Statistical comparisons were made by ANOVA with a Bonferroni post hoc test for multiple comparisons test. An analysis of variance with repeated measurements (ANOVA) was used to test concentration-dependent changes in afferent arteriolar diameter and to assess the differences in the afferent arteriolar responses, t-Test and One-Way ANOVA followed by Student-Newman-Keuls post hoc test were used for other comparisons among groups. All statistical calculations were made using Graphpad Prism (6.01, La Jolla, CA, USA). *P*-value < 0.05 was accepted as statistically significant.

## Results

There were no differences in food and water intake, body weight and kidney weight among all groups. However, blood glucose was dramatically higher in diabetic mice (20.7 ± 1.0 versus 6.7 ± 0.3 mmol.l^−1^, *P*<0.001), Administration of sulindac did not affect the blood glucose levels in either non-diabetic or diabetic mice (Table 1).

### Enhanced ET-1 responses of afferent arterioles are associated with excessive ROS in diabetic mice

**Fig. 1.**
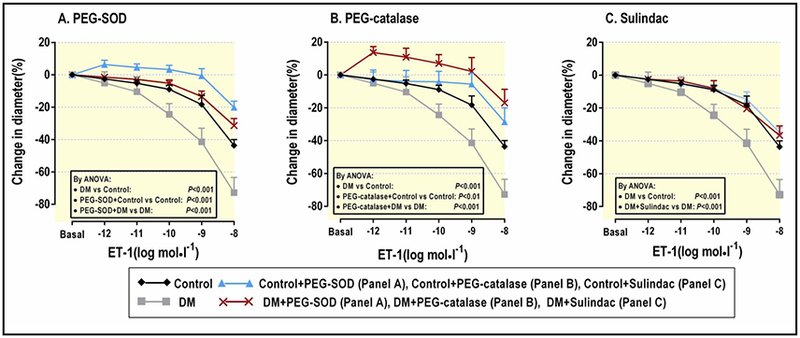
Meant ± SEM values (n =5–6) for ET-1 responses of afferent arterioles from control mice in the absence (black diamonds with black line) or presence of PEG-SOD (200 units mL^−1^) (A) or PEG-catalase (1000 units mL^−1^) (B) or Sulindac (40 mg·kg^−1^ day^−1^ for 4 weeks) (C) (blue triangles with blue line), diabetic mice in the absence (grey squares with grey line) or presence of PEG-SOD (A) or PEG catalase (B) or Sulindac) (C) (red crosses with red line). Data are shown for changes in luminal dimeters of afferent arterioles in response to ET-1 (10^−12^ - 10^−8^ mol·l^−1^). ANOVA, analysis of variance; ET-1, endotheline-1; PEG, polyethylene glycol; SOD, superoxide dismutase; DM, diabetes mellitus.

**Fig. 2.**
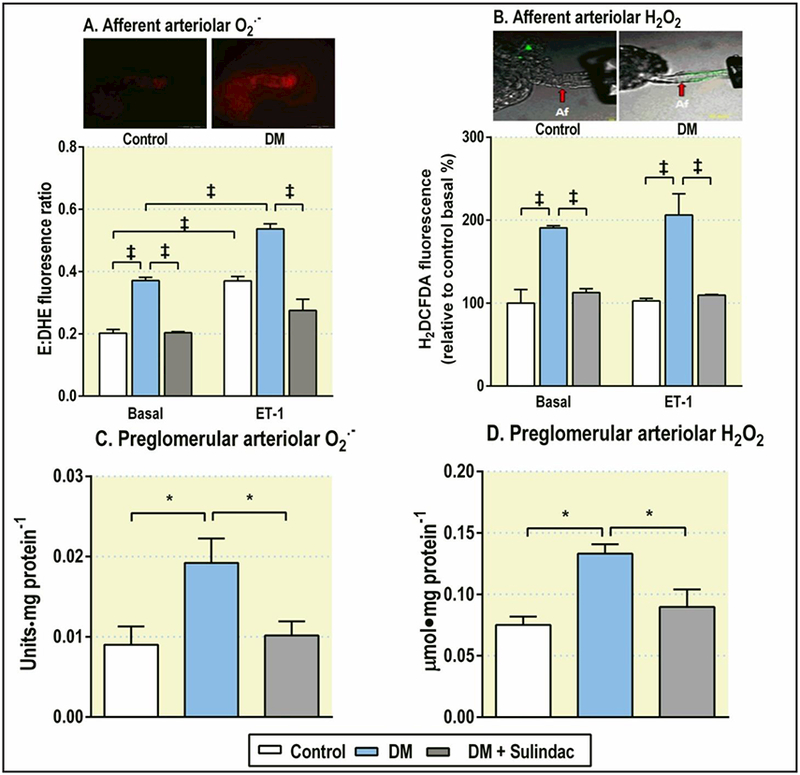
Mean ± SEM values (n=5) for O_2_^.−^ and H_2_O_2_ in the perfused individual afferent arteriole (A and B) or in the isolated preglomerular arterioles (C and D) from control mice (open boxes), diabetic mice (blue filled boxes) and sulindac treated diabetic mice (grey filled boxes). Data are shown as the basal levels of ethidium:dihydroethidium (E:DHE) fluorescence ratio for O_2_^.−^, H_2_DCFDA fluorescence for H_2_O_2_, their changes in response to ET-1 (10^−8^ mol·l^−1^) in the perfused individual arteriole and units (or μmol)·mg-l protein for their concentrations in the isolated preglomerular arterioles. Comparing groups: * P<0.05; ‡ P< 0.001.

**Fig. 3.**
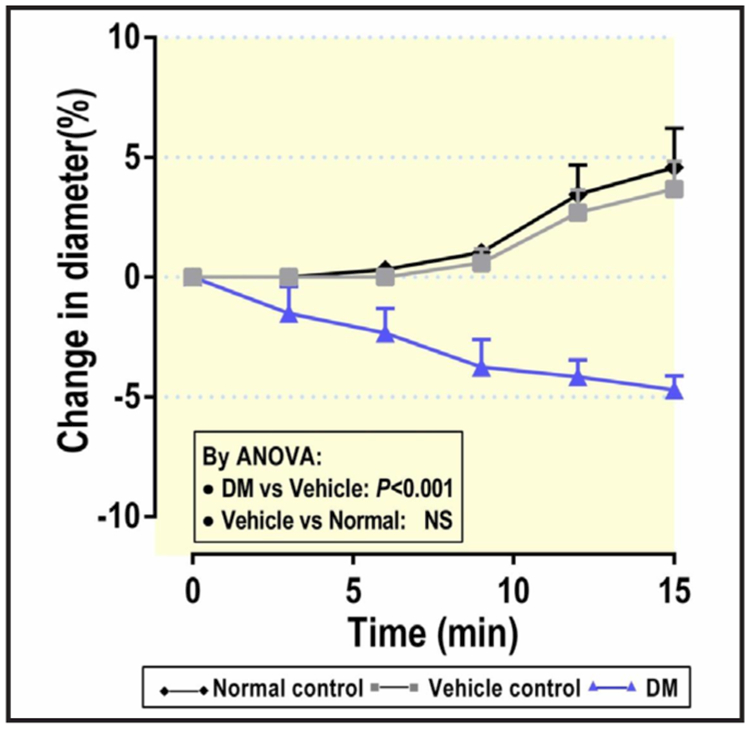
Time course of changes in luminal diameter of perfused afferent arterioles (n=5) from normal control (without injection, black diamonds with solid line), vehicle control (7 days after injection of citrate buffer, grey diamonds with grey line) and diabetic mice (7 days after injection of STZ, blue triangles with blue line) by bath addition of H_2_O_2_ (10 μmol·l^−1^). ANOVA, analysis of variance.

Afferent arterioles from diabetic mice had increased responses to ET-1 at 10^−8^ mol·l^−1^ (DM: −72 ± 4% versus control: −43 ± 4%, *P*<0.05) (Fig. 1), Compared to controls, the ethidium:dihydroethidium (E:DHE) fluorescence ratio (probe for O_2_^.−^) and the 6-carboxy-2′, 7′-dichlorodihydrofluorescein diacetate (H_2_DCFDA) fluorescence (probe for H_2_O_2_) were increased in the perfused afferent arterioles from diabetic mice (Fig. 2A and B), E:DHE ratio increased (Fig. 2A) whereas H_2_DCFDA fluorescence levels similar to their basal levels (Fig. 2B) after incubation with ET-1 both in normal and diabetic arterioles. O_2_^.−^ and H_2_O_2_ concentrations in the isolated preglomerular arterioles were increased in diabetic animals (Fig. 2C and D). Both measurements, fluorescence and biochemical determination, suggest increased O_2_^.−^ and H_2_O_2_ concentrations in arterioles of diabetic mice compared to controls. Bath addition of PEG-SOD (200 units·ml^−1^, 30 min) reduced the responses to ET-1 in both control and diabetic mouse arterioles (Fig. 1A), On the other hand, bath addition of PEG-catalase (1000 units·ml^−1^, 30 min) substantially reduced the responses to ET-1 selectively in arterioles from diabetic mice (Fig. 1B), In consistent to the vasodilatory responses to H_2_O_2_ in normal afferent arterioles [19], incubation of afferent arterioles with H_2_O_2_ (10 μmol·l^−1^, 15min) led to a similar vasodilation in vehicle controls (by ANOVA, *P*=NS). However, a slowly developing vasocontraction to H_2_O_2_ was observed in diabetic mice (by ANOVA, *P*<0.001, Fig. 3).

### Canonical Wnt signaling is enhanced in diabetic mice

**Fig. 4.**
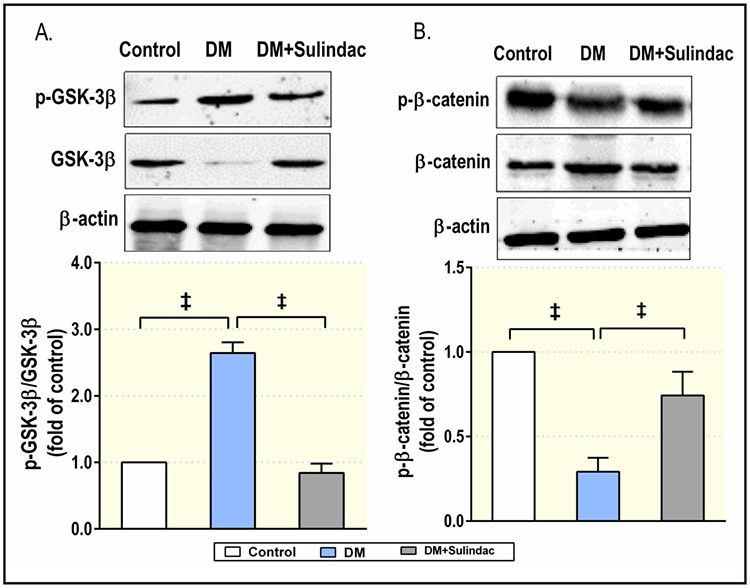
Mean ± SEM values (n=5) for the ratio of phosphorylated protein p-GSk-3β/GSk-3β (A) and p-β-catenin/β-catenin (B) in preglomerular arterioles from control mice (open boxes), diabetic mice (blue filled boxes) and Sulindac treated diabetic mice (grey filled boxes). Comparing groups: * P< 0.05; ‡ P< 0.001.

Canonical Wnt signaling was activated in renal microvessels from diabetic mice, as evidenced by a 2.6-fold increase in protein expression for p-GSK-3β/GSK3β ratio (Fig. 4A) and a 3.3-fold decrease in p-β-catenin/β-catenin ratio (Fig. 4B), These changes were restored to normal in the preglomerular arterioles from diabetic mice treated with sulindac to inhibit Wnt signaling (Fig. 4).

### Expressions and activities of catalase and S0D2 are decreased in renal microvessels from diabetic mice

**Fig. 5.**
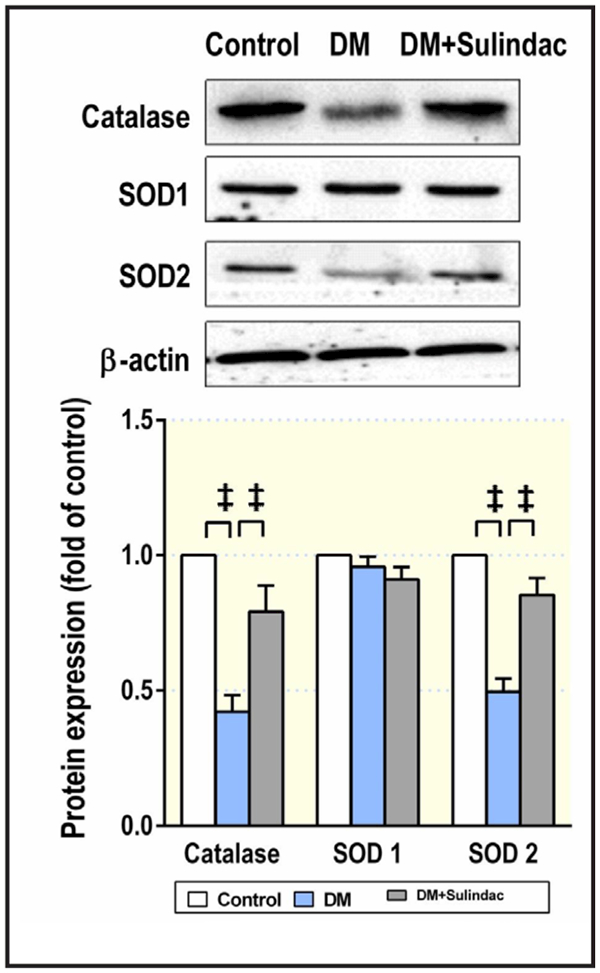
Mean ± SEM values (n=5) for protein expression of catalase, SOD1 and SOD2 in preglomerular arterioles from control mice (open boxes), diabetic mice (blue filled boxes) and Sulindac treated diabetic mice (grey filled boxes). Comparing groups: ‡ P< 0.001.

**Fig. 6.**
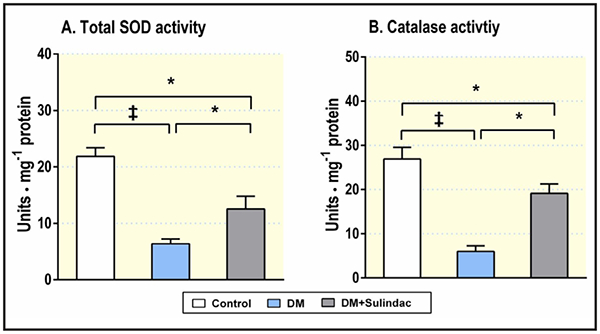
Mean ± SEM values (n=5) for enzymatic activity of total SOD (A) and catalase (B) in preglomerular arterioles from control mice (open boxes), diabetic mice (blue filled boxes) and Sulindac treated diabetic mice (grey filled boxes). Comparing groups: * P< 0.05; ‡ P< 0.001.

The expression of catalase and SOD2 were decreased in renal microvessels from diabetic mice, but these effects were prevented by sulindac (Fig. 5), There were parallel reductions in catalase and total SOD activities in diabetic mice and were improved by treatment with sulindac (Fig. 6).

### Sulindac normalizes the ET-1 responses of afferent arterioles through reduction of ROS in diabetic mice

Given sulindac to mice for 4 weeks, simultaneously lowered the E:DHE ratio and H_2_DCFDA fluorescence (Fig. 2A and B) as well as O_2_^.−^ and H_2_O_2_ concentrations (Fig. 2C and D) and normalized the ET-1 responses of afferent arterioles in diabetic mice. However, it did not affect the ET-1 responses (Fig. 1C) or ROS generation (data not shown) of afferent arterioles in non-diabetic mice. Therefore, in the following Figures (2, 4, 5, 6), we did not show its effects in non-diabetic setting.

## Discussion

The main finding of the present study is that diabetes enhances the responses to ET-1 and the increases of O_2_^.−^ and H_2_O_2_ substantially in renal afferent arterioles. The arteriolar O_2_^.−^ was increased in control and increased further in diabetic mice by incubation with maximal concentrations of ET-1. These changes of O_2_^.−^ and H_2_O_2_ in diabetic mice were all prevented in those treated with sulindac that was confirmed to inhibit Wnt signaling from the reversal of expression of Wnt signaling proteins. The increased O_2_^.−^ in diabetic mouse arterioles, and its prevention by sulindac, was accompanied by decreased protein expression for SOD2 and decreased total SOD activities whereas the corresponding changes in H_2_O_2_ were accompanied by similar changes in catalase. Incubation of arterioles from control or diabetic mice with PEG-SOD attenuated their responses to ET-1 whereas incubation with PEG-catalase markedly attenuated the responsiveness to ET-1 selectively in diabetic mice. The enhanced ET-1 response of individual afferent arteriole from diabetic mice extends previous reports in microvessels [8, 24] or renal arteries [25].

Oxidative stress contributes to diabetic vasculopathy in animal models and in particular with diabetic nephropathy [26], We confirmed increases of O_2_^.−^ and H_2_O_2_ in renal arterioles from diabetic mice. O_2_^.−^ is formed enzymatically or in mitochondria by the univalent reduction of oxygen, its major route of metabolism is to H_2_O_2_ via SOD [27], The H_2_O_2_ level is regulated by various peroxidase of which catalase is most important in small vessels, for its abundance and higher affinity to H_2_O_2_. Thus, we evaluated arteriolar protein levels and activities of SODs and catalase separately to assess the extent of oxidative stress, for their important roles in regulation of cellular ROS. We reported previously that deficiency in SOD1, SOD2 or SOD3 had dramatically increased afferent arteriolar level of O_2_^.−^ and enhanced contractility [28], Transfer of SOD1 or SOD2 gene to the diabetic vessels decreased O_2_^.−^ and improved vascular function [29, 30], We confirmed the reduced expression of SOD2 in arterioles from diabetic mice, but expression of SOD1 was maintained. The reduction in SOD2 may underline the finding that mitochondria are the main site of producing superoxide in diabetes [31, 32], Since SOD2 plays a central role in metabolizing O_2_^.−^ in mitochondria the maintained level of SOD1 may prevent O_2_^.−^ overproduction in diabetic conditions. Interestingly, SOD activity in vascular beds showed an increase [33] or decrease [34, 35] in different animal models of diabetes. Likewise, vascular catalase activity or protein level also have been reported to be increased or decreased in diabetes [34, 36, 37], These inconsistent results perhaps depend on the stage or severity of the diabetic model. Our study clearly demonstrates a comprised antioxidant SOD and catalase system in the vasculature of diabetic mice that could contribute to the increases in O_2_^.−^ and H_2_O_2_. However, our key findings need further confirmation in other non-toxic diabetes model.

Previously studies have reported that ET-1 modulates renal arteriolar tone via generation of O_2_^.−^ [38]. Our study confirmed that metabolism of O_2_^.−^ with PEG-SOD reduced ET-1 responses in both control and diabetic mice. Afferent arteriolar levels of O_2_^.−^ were increased significantly by ET-1 at 10^−7^mol·l^−1^ in arterioles from both normal and diabetic mice. Thus, generation of O_2_^.−^ contributes to ET-1 induced arteriolar contractions in both normal and diabetic mouse arterioles. Compared to O_2_^.−^, H_2_O_2_ has been reported variously to exert vasoconstriction or vasodilation depending on experimental conditions [5, 39], We confirmed our previous report that 10^−5^ mol·l^−1^ of H_2_O_2_ vasodilates normal afferent arterioles [19]. In contrast, we now report that H_2_O_2_ exerts a steady and sustained contraction of afferent arterioles from diabetic mice. The mechanisms of these different effects are unclear. The possible mediators are increased contractile protein expression [40] or decreased potassium channels protein [6,41]. We confirmed that metabolism of H_2_O_2_ with PEG-catalase did not affect contractions of arterioles from normal mice [7], However, PEG-catalase markedly reduced the responsiveness to ET-1 in arterioles from diabetic mice. Increased H_2_O_2_ was related to diabetes rather than ET-1 since it was found in diabetic arterioles under basal condition and was not increased even by 10^−7^ mol·l^−1^ of ET-1. This is the first report that increased ROS generated by diabetes, especially H_2_O_2_, are responsible for the increased responses to ET-1.

Canonical Wnt signaling inactivates GSK-Ββ, thereby stabilizing the arterial transcription factor β-catenin that activates many target genes,[42] notably that implicated in the metabolic syndrome and diabetes [43, 44], We detected activation of the canonical Wnt pathway with increased β-catenin and decreased GSK-Ββ in preglomerular arterioles from diabetic mice that were corrected by sulindac. This effect was in confirmed with previous reports that even though sulindac is a commonly used non-steroidal anti-inflammatory drug, it also significantly suppressed Wnt/β-catenin signaling [45], Oxidative stress has been reported variously to activate [46] or inhibit [47] canonical Wnt signaling. However, our data confirmed other reports that Wnt/β-catenin pathway promotes ROS generation [48–50], We found that administration of sulindacin diabetic mice decreased H_2_O_2_ and O_2_^.−^ and increased catalase and SOD2 expression and activities in preglomerular arterioles. We noticed that the activities of arteriolar total SOD and catalase recovered partially with more pronounced reduction of ROS levels after administration of sulindac to diabetic mice. Moreover, sulindac also blunted O_2_^.−^ and H_2_O_2_ generation induced by ET-1 in diabetic mice. This indicates the important roles for sulindac in reducing microvascular ROS in diabetes. Overexpression of β-catenin in the endothelium also increased ROS and led to vascular dysfunction [13]. Our data indicate that activation of canonical Wnt signaling is essential for excessive vascular ROS in diabetes mellitus and point to reduction in the ROS metabolism pathways as major targets. In this study, we acknowledge some limitations. First, the plasma creatinine was not measured in this study, thus it is unclear whether the drug had such an effect in STZ-induced diabetic mice. Second, sulindac used in this study is not a perfect inhibitor for canonical Wnt signaling.

## Conclusion

Present study provides an evidence that diabetes leads to activation of canonical Wnt signaling that enhances renal afferent arteriolar response to ET-1 through promoting ROS, notably H_2_O_2_. Excessive ROS contributed to the enhanced responses to ET-1 that may relate to reduced catalase and SOD2 expression. Blockade of canonical Wnt signaling *in vivo* were concomitant with restored SOD2 and catalase and normalized the arteriolar responses to ET-1. Therefore, antioxidant strategies targeting canonical Wnt signaling may correct abnormal vascular function in diabetic nephropathy.

## Figures and Tables

**Table 1.** Blood glucose levels (mmol.l^−1^) in non-diabetic and diabetic mice. Diabetic vs Non-diabetic: P < 0.001; Non-diabetic + Sulindac vs Non-diabetic: NS; Diabetic + Sulindac vs Diabetic: NS

Mice	Mean ± SEM	N	T-test
Non-diabetic	6.7 ± 0.3	8	
Diabetic	20.7 ± 1.0	17	P < 0.001
Non-diabetic + Sulindac	6.9 ± 0.3	8	NS
Diabetic + Sulindac	21.4 ± 0.9	16	NS
